# Genome-Wide Association Mapping for Female Infertility in Inbred Mice

**DOI:** 10.1534/g3.116.031575

**Published:** 2016-07-21

**Authors:** Ji-Long Liu, Tong-Song Wang, Miao Zhao

**Affiliations:** * College of Veterinary Medicine, South China Agricultural University, Guangzhou 510642, China; † Department of Biology, Shantou University, Shantou 515063, China

**Keywords:** Genome-wide association mapping, Female infertility, Inbred mice, Decidualization

## Abstract

The genetic factors underlying female infertility in humans are only partially understood. Here, we performed a genome-wide association study of female infertility in 25 inbred mouse strains by using publicly available SNP data. As a result, a total of four SNPs were identified after chromosome-wise multiple test correction. The first SNP rs29972765 is located in a gene desert on chromosome 18, about 72 kb upstream of *Skor2* (SKI family transcriptional corepressor 2). The second SNP rs30415957 resides in the intron of *Plce1* (phospholipase C epsilon 1). The remaining two SNPs (rs30768258 and rs31216810) are close to each other on chromosome 19, in the vicinity of *Sorbs1* (sorbin and SH3 domain containing 1). Using quantitative RT-PCR, we found that *Sorbs1* is highly expressed in the mouse uterus during embryo implantation. Knockdown of *Sorbs1* by siRNA attenuates the induction of differentiation marker gene *Prl8a2* (decidual prolactin-related protein) in an *in vitro* model of decidualization using mouse endometrial stromal cells, suggesting that *Sorbs1* may be a potential candidate gene for female infertility in mice. Our results may represent an opportunity to further understand female infertility in humans.

The global prevalence of human infertility is about 9% (Boivin *et al.* 2007). In 38% of all infertility cases, the predominant cause is female infertility, which is defined as the inability of a female to cause pregnancy with a fertile male (de Kretser 1997). Several studies have revealed that genetic factors contribute to female infertility (Matzuk and Lamb 2008). It has been shown that SNPs in tumor-suppressor p53 and its family members (p63 and p73) are associated with recurrent implantation failure in humans (Kang *et al.* 2009; Feng *et al.* 2011). Moreover, the SNP rs6788044 in PTX3 (pentraxin 3) is associated with higher numbers of offspring (May *et al.* 2010). Notably, a significant association between rs10846744 SNP in SCARB1 (scavenger receptor class B member 1) and clinical pregnancy rate was found in the African-American population but not in the Caucasian group, suggesting an ethnic bias (Yates *et al.* 2011). Recently, a genome-wide association study has identified a single SNP (rs100009124, in a gene desert on chromosome 4) associated with number of pregnancies at *P* < 10^−6^, and with number of children at *P* < 10^−7^ (Aschebrook-Kilfoy *et al.* 2015). Despite these discoveries, the genetic factors underlying female infertility in humans are only partially understood.

In recent years, with the development of high-throughput genotyping technologies, databases containing genome-wide genetic variations for common inbred mouse strains have been created (Bennett *et al.* 2010; Mott 2007). Because all mice from an inbred strain are genetically identical and homozygous, these databases in conjunction with phenotypic information on corresponding strains open the opportunity for genome-wide association mapping of quantitative traits in an easy and cost-effective way (Flint and Eskin 2012). So far, this approach has been used successfully in numerous studies (Webb *et al.* 2009; Johnson *et al.* 2012; Paun and Haston 2012; Davis *et al.* 2013; Himes *et al.* 2013; Hadsell *et al.* 2015).

In the present study, we analyzed the degree of phenotypic variation in female infertility across 25 inbred mouse strains. Our results may represent an opportunity to further understand female infertility in humans.

## Materials and Methods

### Phenotypic and genotypic data

Phenotypic data were obtained from the Mouse Phenome Database at Jackson Laboratory (http://phenome.jax.org/). Female infertility was measured as the percent of matings that were nonproductive (MPD:14934) in 33 inbred mouse strains. The phenotypic data were log transformed. Genotypic data were downloaded from mouse HapMap database at Broad Institute (http://www.broadinstitute.org/mouse/hapmap/). A total of 132,285 SNPs for 25 out of the 33 inbred mouse strains with available female infertility data were retrieved. The 25 inbred mouse strains were: 129P3/J, 129X1/SvJ, A/J, AKR/J, BALB/cByJ, BALB/cJ, C3H/HeJ, C3H/HeOuJ, C3HeB/FeJ, C57BL/10J, C57BL/6J, C57BLKS/J, C57L/J, CBA/CaJ, CBA/J, DBA/1J, DBA/1LacJ, DBA/2J, LP/J, NZB/BlNJ, NZW/LacJ, PL/J, SJL/J, SM/J, and SWR/J. The SNPs with > 10% of missing genotype calls were removed.

### Genome-wide association mapping

To determine the association between SNPs and female reproductive traits, we applied a weighted F-test. The use of a single SNP is restrictive in the sense that it allows a representation of the genome only as diallelic. The use of windows of multiple SNPs enables the visualization of more complex genomic relationships between multiple strains. A window of three SNPs was used as described previously (Pletcher *et al.* 2004). For each three-SNP window, let the haplotype group be *g*. We assumed that the total number of mouse strains is *N*, and the total number of haplotypes is *k*. The female reproductive trait vector is denoted as *x_i_* (*i* = 1,2,…,*N*). The weighted F-statistic has the following form:

$$\text{F=}\frac{{\text{MS}}_{\text{BG}}}{{\text{MS}}_{\text{WG}}}$$

$${\text{MS}}_{\text{BG}}\text{=}\frac{{\text{SS}}_{\text{BG}}}{df_{\text{BG}}}=\frac{\sum_{g}\omega_{\text{X}}n_{g}{\left(\mu_{g}-\mu_{\text{T}}\right)}^{2}}{k-1}$$

$${\text{MS}}_{\text{WG}}\text{=}\frac{{\text{SS}}_{\text{WG}}}{df_{\text{WG}}}=\frac{\sum_{g}\sum_{i=1}^{n_{g}}\omega_{g}{\left(x_{g_{i}}-\mu_{g}\right)}^{2}}{N-k}$$

Where *μ_g_* is the mean of phenotypic values in a given inferred haplotype, *μ*_T_ is the mean of all phenotypic values. The genetic diversity between two strains is defined as the number of disagreed SNPs genome-wide divided by the total number of SNPs under consideration. The *ω_g_* in the weighted F-statistic is the average genetic diversity between all strain pairs contained in the inferred haplotype. We calculated chromosome-wise thresholds for multiple testing using Bonferroni multiple test correction. In this way, the family-wise error rate is 1 – (1 – α*_i_*)*^n^* ≈ α*_i_n* where α*_i_* is the individual test rejection level, and *n* is the number of SNPs on the chromosome under consideration. The weighted F-test algorithm was implemented in the MATLAB computing environment (MathWorks, Natick, MA).

### Gene set enrichment analysis

For gene set enrichment analysis, we adopted the classification terms defined by MGI GOslim (http://www.informatics.jax.org/gotools/MGI_GO_Slim.html). The ontology covers three categories: biological process, cellular component, and molecular function. The Gowinda software (Kofler and Schlotterer 2012) was used to test for the enrichment of particular terms under the biological process category. Significant SNPs (*P* < 0.001) were used as the query set, and all remaining SNPs used in the genome-wide mapping were treated as the background SNP set. The Gowinda software was run with the following parameters:–simulations 100000–gene-definition updownstream10000–mode gene–min-genes 1. In the end, false discovery rate (FDR) < 0.05 was used as significance threshold to identify enriched terms.

### Mouse model of early pregnancy

A mouse model of early pregnancy was established using CD1 outbred mice. The CD1 mice were first produced by Charles River Laboratories in 1959 (Rice and O’Brien 1980). They can be traced back directly to Webster’s Swiss mice from the Rockefeller Institute. CD1 mice are used widely in general purpose biomedical researches. Mature CD1 mice were housed in a temperature- and light-controlled environment (14-hr light/10-hr dark) with free access to regular food and water. Female mice were mated with fertile males to induce pregnancy. Uterine fragments from the implantation site (IS) and the interimplantation site (IIS) were collected separately on d 5 and 8 of pregnancy (d 1 = day of vaginal plug). On d 5 of pregnancy, the IS of the uterus was visualized through intravenous injection of 0.1 ml of 1% Chicago blue dye (Sigma-Aldrich, St. Louis, MO) in saline. The embryo at IS was removed by flushing the uterine horn with saline, and successful removal was confirmed by examining the recovered embryos. On d 8 of pregnancy, embryonic tissues from IS segments were dissected out under a stereomicroscope. In order to ensure complete removal, the IS segments was cut into half and one half was examined in sequential frozen sections. Samples were flash-frozen in liquid nitrogen and stored at –80° until use. All animal procedures were approved by the Institutional Animal Care and Use Committee of South China Agricultural University.

### Isolation of mouse endometrial stromal cells

The uterus from CD1 mice was split longitudinally on d 4 of pregnancy. Luminal epithelial cells were removed by digestion in HBSS containing 1% trypsin (AMRESCO Inc., Solon, OH) and 6 mg/ml dispase (Roche Diagnostics GmbH, Mannheim, Germany). The remaining tissues were incubated with 0.15 mg/ml collagenase I (Invitrogen). The supernatants were filtrated through 70 μm wire gauze, and centrifuged to collect the stromal cells. The cell pellets were washed twice with HBSS, and resuspended in complete DMEM/F-12 medium (Sigma-Aldrich) with 10% charcoal-treated fetal bovine serum (cFBS, Invitrogen, Carlsbad, CA). Cells were seeded onto 24-well culture plates at a concentration of 2 × 10^5^ cells/well. After an initial 30-min culture, cells were further cultured in fresh medium with 2% cFBS.

### Transfection and in vitro decidualization

The siRNAs targeting *Sorbs1* were transfected into isolated mouse endometrial stromal cells using Lipofectamine 2000 (Invitrogen). Nonspecific scramble siRNA was transfected as a negative control. The medium was replaced with complete culture medium containing 2% cFBS 6 hr after transfection. Following an additional 12 hr of culture, cells were *in vitro* decidualized with estrodiol-17β (10 nM, Sigma-Aldrich) and progesterone (1 μM, Sigma-Aldrich), respectively. Cells were harvested for further analysis after 48 hr.

### Quantitative RT-PCR

The total RNA from mouse uterus or cultured cells was isolated by using TRIzol reagent (Invitrogen). Potential DNA contamination was eliminated by digestion with RQ1 deoxyribonuclease I (Promega, Madison, WI). Reverse transcription was performed with the PrimeScript reverse transcriptase reagent kit (Takara, Dalian, China). For quantitative RT-PCR, cDNA was amplified using a SYBR Premix Ex Taq kit (TaKaRa) on the Rotor-Gene 3000A system (Corbett Research, Mortlake, Australia). *Rpl7* was used as a reference gene for normalization. Data were analyzed using the 2^–ΔΔCt^ method. All of the experiments were repeated independently at least three times. The significance of difference between two groups was assessed by Student’s *t*-test. Results are expressed as mean ± SEM. *P* < 0.05 was considered statistically significant.

The primer sequences were:

*Sorbs1*-forward: 5′-TTTTCCAGGCAACTATGT-3′;
*Sorbs1*-reverse: 5′-ATGCTTCATCCTCCGATA-3′.
*Prl8a2*-forward: 5′-AGCCAGAAATCACTGCCACT-3′;
*Prl8a2*-reverse: 5′-TGATCCATGCACCCATAAAA-3′.
*Rpl7*-forward: 5′-GCAGATGTACCGCACTGAGATTC-3′;
*Rpl7*-reverse: 5′-ACCTTTGGGCTTACTCCATTGATA-3′.

### Data availability

The authors state that all data necessary for confirming the conclusions presented in the article are represented fully within the article.

## Results

### Analysis of female infertility data among inbred mouse strains

**Figure 1:**
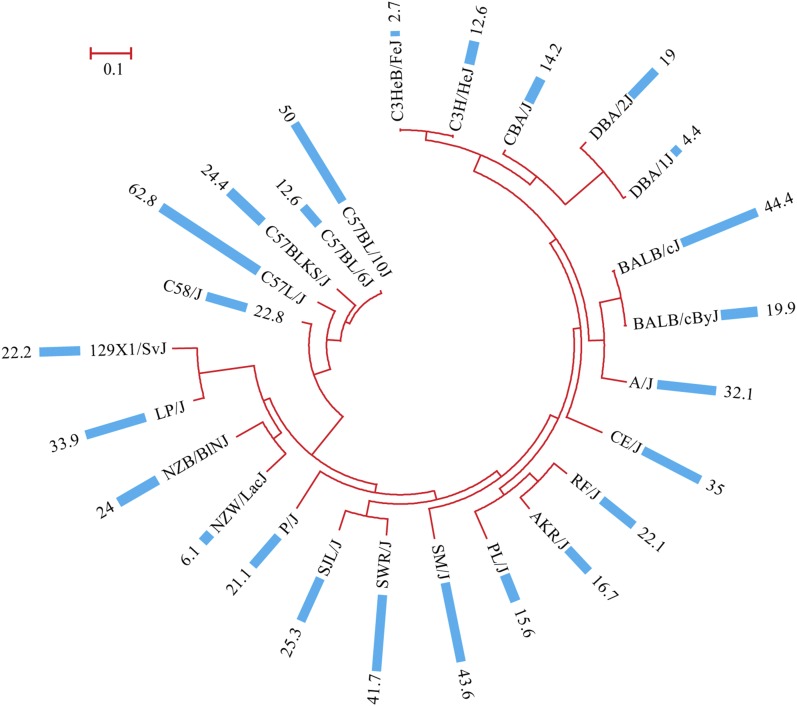
Female infertility data measured as percent of nonproductive matings among 25 mouse strains along with the phylogenic tree based on SNP data. The maximum-likelihood phylogenic tree was built using the pseudoalignment of all SNP alleles for 25 strains with the PhyML package version 3.0. Percent of nonproductive matings is shown as a bar plot. The exact value is shown as a label at the right side of each bar.

In the present study, we retrieved phenotypic data from the Mouse Phenome Database at Jackson Laboratory (http://phenome.jax.org/). Female infertility was measured as the percent of matings that were nonproductive in 25 inbred mouse strains. The female infertility data along with a phylogenic tree built from the SNP data of all 25 strains are shown in Figure 1. Using an arbitrary cutoff of 20%, 15 strains (P/J, RF/J, 129X1/SvJ, C58/J, NZB/BlNJ, C57BLKS/J, SJL/J, A/J, LP/J, CE/J, SWR/J, SM/J, BALB/cJ, C57BL/10J, and C57L/J) were of high female infertility, whereas 10 strains (C3HeB/FeJ, DBA/1J, NZW/LacJ, C57BL/6J, C3H/HeJ, CBA/J, PL/J, AKR/J, DBA/2J, and BALB/cByJ) were of low female infertility. The strain with the lowest female infertility was C3HeB/FeJ (2.7%), whereas the strain with the highest female infertility was C57L/J (62.8%).

### Genome-wide association mapping

**Figure 2:**
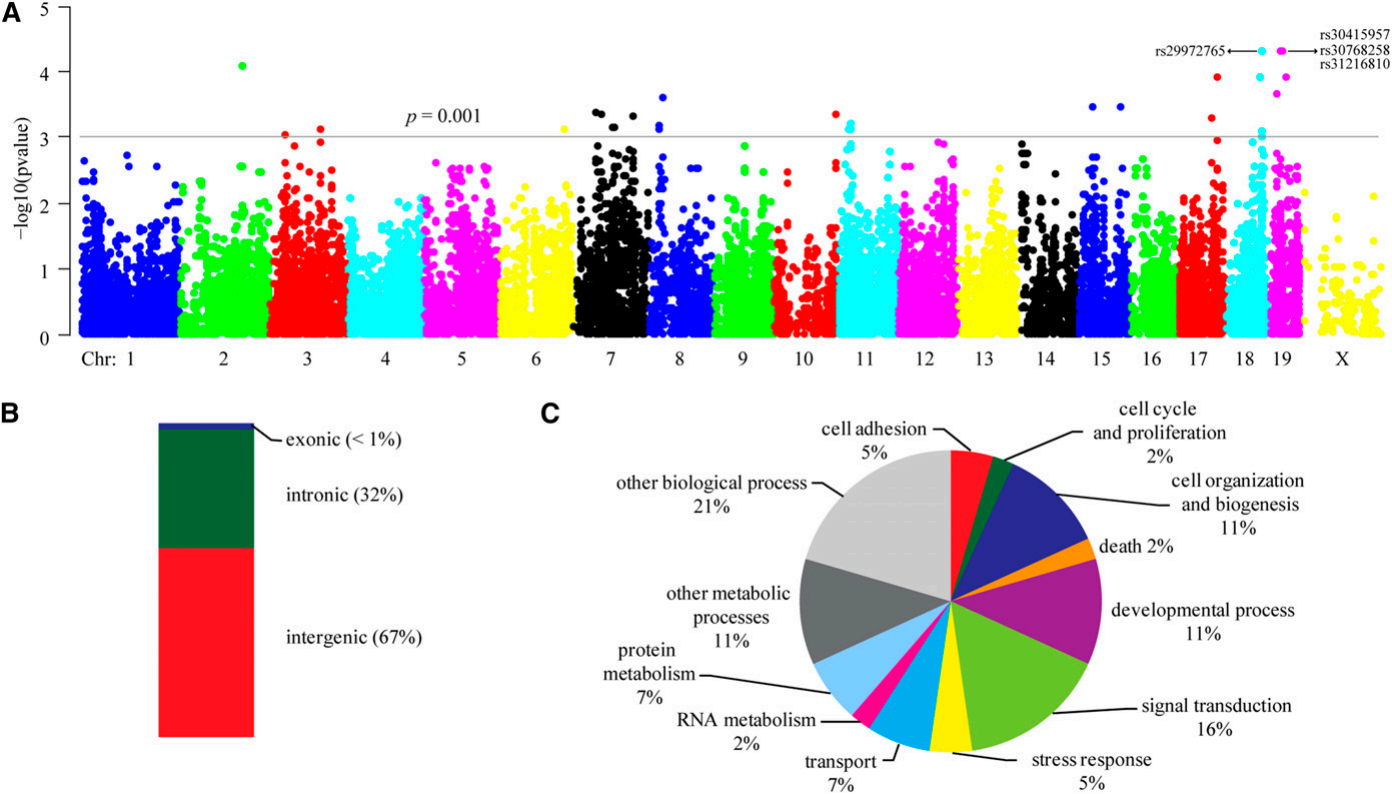
Genome-wide association mapping of female infertility in mice. (A) Manhattan plot for genome-wide association mapping of female fertility. Female fertility was measured in the term of percent of nonproductive mating. Genomic location is shown on the *x*-axis. Genome-wide association analysis was conducted by using weighted F-test. The horizontal line at *P* = 0.001 is the threshold for genome-wide significance. The four SNPs which are significant (*P* < 0.05) after chromosome-wide Bonferroni multiple test correction are labeled on the plot. (B) Composition of significant SNPs (*P* < 0.001). (C) Gene ontology (GO) enrichment analysis of genes at significant SNPs. The pie diagram demonstrates the percentage of genes within GO biological process categories.

Using the female infertility data for 25 inbred mouse strains, we preformed a genome-wide association mapping analysis. For studies using inbred mouse strains, one of the major problems is the lack of randomness in breeding histories. As shown in the phylogenic tree (Figure 1), subgroups that differ phenotypically may lead to spurious associations (Tian *et al.* 2008). To correct for genetic relatedness among these inbred mouse strains, we employed the weighted F-test using a three-SNP window (detailed in *Materials and Methods*). The association results were used to generate a Manhattan plot (Figure 2A). As a result, we detected 158 SNPs (listed in Supplemental Material, Table S1) that reached the statistically significant threshold (*P* < 0.001).

The location of all significant SNPs relative to annotated genes on the mouse genome was calculated. We found that the vast majority of SNPs were intergenic (67%) or intronic (32%); only one SNP (< 1%) was intragenic, residing on the 3′-UTR, to be exact (Figure 2B). To further characterize these candidate SNPs, we performed gene ontology (GO) term enrichment analysis. Because a long gene tends to contain more false-positive candidate SNPs than a short one, the bias in gene length may result in inaccurate enrichment for a SNP set. We thus employed the Gowinda software, which enables the correction of gene length bias by using a permutation approach. Gowinda was run in the gene mode, and all candidate SNPs within 10,000 bp of a gene were considered. As a result, we identified 12 biological processes for all significant SNPs: cell adhesion (5%), cell cycle and proliferation (2%), cell organization and biogenesis (11%), death (2%), developmental processes (11%), signal transduction (16%), stress response (5%), transport (7%), RNA metabolism (2%), protein metabolism (7%), other metabolic processes (11%), and other biological processes (21%) (Figure 2C). Based on the enrichment test of Gowinda, signal transduction was the only term that was significantly enriched (FDR < 0.05).

### Identification of Sorbs1 as a candidate gene for female infertility

**Figure 3:**
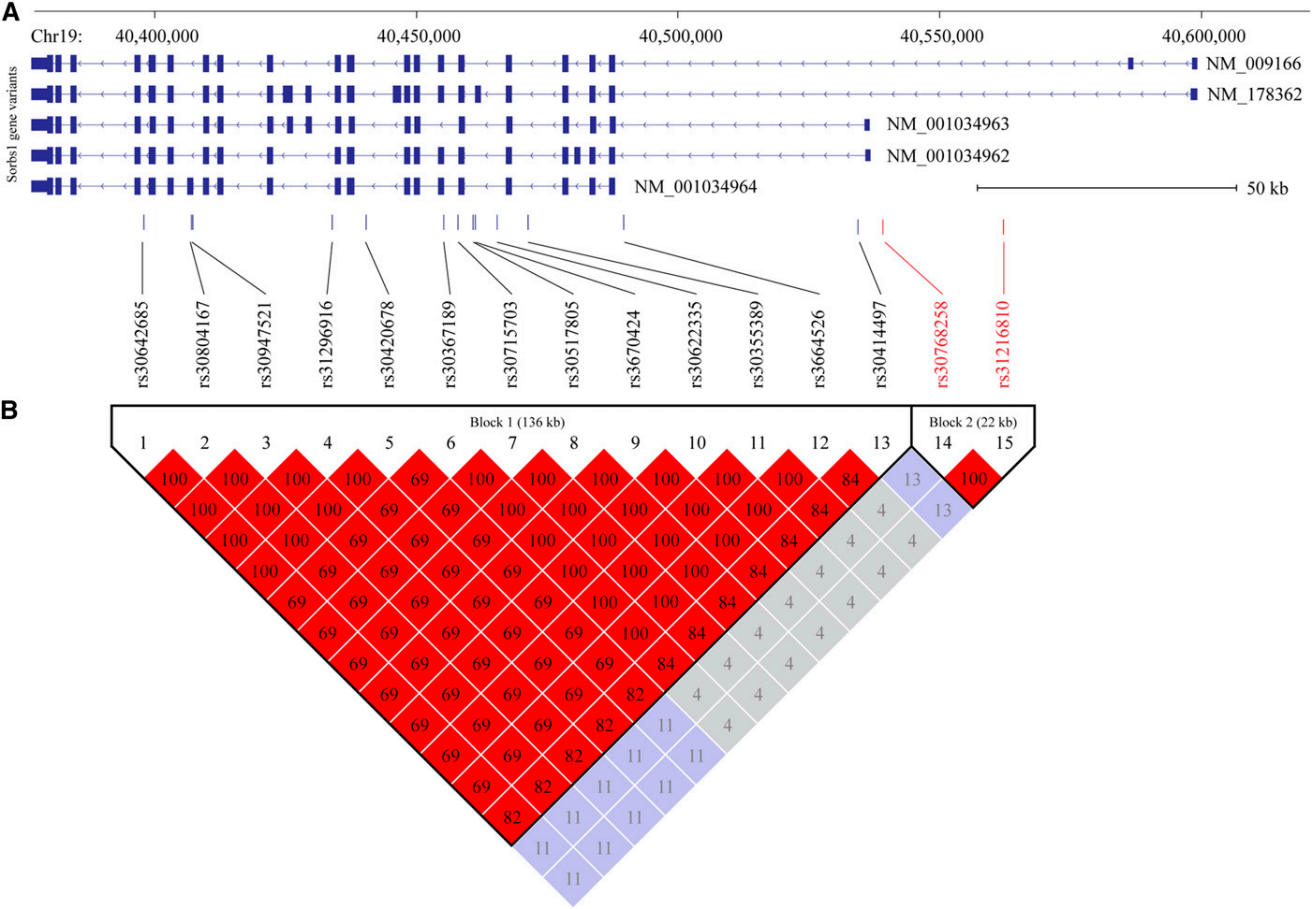
Linkage disequilibrium (LD) structure for the region containing rs30768258 and rs31216810 near *Sorbs1* on chromosome 19. (A) Transcript models for the *Sorbs1* gene locus. Blocks correspond to exons and lines indicate introns. The chevrons within the intronic lines indicate the direction of transcription. SNP sites are shown at the bottom of the transcript models. The two significant SNP sites were colored red. (B) LD blocks on the Sorbs1 gene locus. The average *r*^2^ for SNP pairs was calculated, and is presented using Haploview software.

In order to select the strongest associations, a chromosome-wide Bonferroni-corrected *P*-value was calculated. Finally, a total of four SNPs (rs29972765, rs30415957, rs30768258, and rs31216810) were still significant after chromosome-wide Bonferroni multiple test correction (*P* < 0.05) (Figure 2A). The first SNP rs29972765 is located on chr18:77022376, a gene desert about 72 kb upstream of *Skor2* (SKI family transcriptional corepressor 2). The second SNP rs30415957 resides on chr19:38799731, in the intron of *Plce1* (phospholipase C epsilon 1). The two remaining SNPs (rs30768258 and rs31216810) are close to each other on chromosome 19 in the vicinity of *Sorbs1* (sorbin and SH3 domain containing 1) (Figure 3A). The linkage disequilibrium (LD) pattern of genomic region surrounding rs30768258 and rs31216810 was investigated by using the Haploview software (Barrett *et al.* 2005). The *r*^2^ values were calculated for surrounding SNPs and a cutoff value of 0.8 as used to set the size of the LD block. This analysis indicated that rs30768258 and rs31216810 have a generally high LD (Figure 3B).

**Figure 4:**
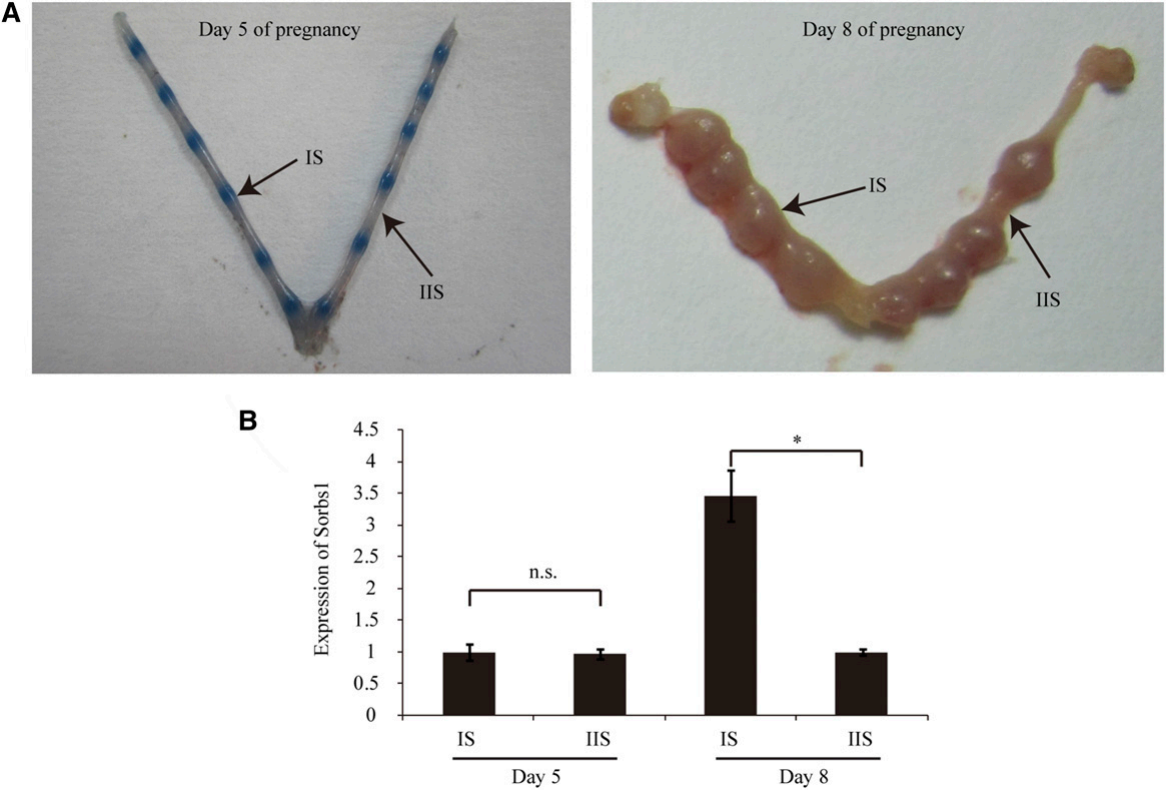
Expression of Sorbs1 in the mouse uterus during embryo implantation. (A) A view of mouse uterus on d 5 and 8 of pregnancy. (B) Quantitative RT-PCR analysis of *Sorbs1* mRNA expression in mouse uterus on d 5 and 8 of pregnancy. IS, implantation site; IIS, interimplantation site; n.s., not significant. * *P* < 0.05.

A previous study has shown that *Sorbs1* is significantly upregulated at implantation sites compared to nonimplantation sites in mouse uterus on d 6.5 of pregnancy (Moreno-Moya *et al.* 2015), suggesting a role of *Sorbs1* during early pregnancy in the uterus. We established a pregnancy model in C57BL/6J mice and quantitative RT-PCR was performed to quantify mRNA expression of *Sorbs1* in mouse uterus during early pregnancy (Figure 4A). There was no significant change in *Sorbs1* mRNA expression between implantation sites and interimplantation sites on d 5 of pregnancy. However, on d 8 of pregnancy, the expression level of *Sorbs1* increased 3.4-fold at the implantation sites compared with interimplantation sites (Figure 4B).

**Figure 5:**
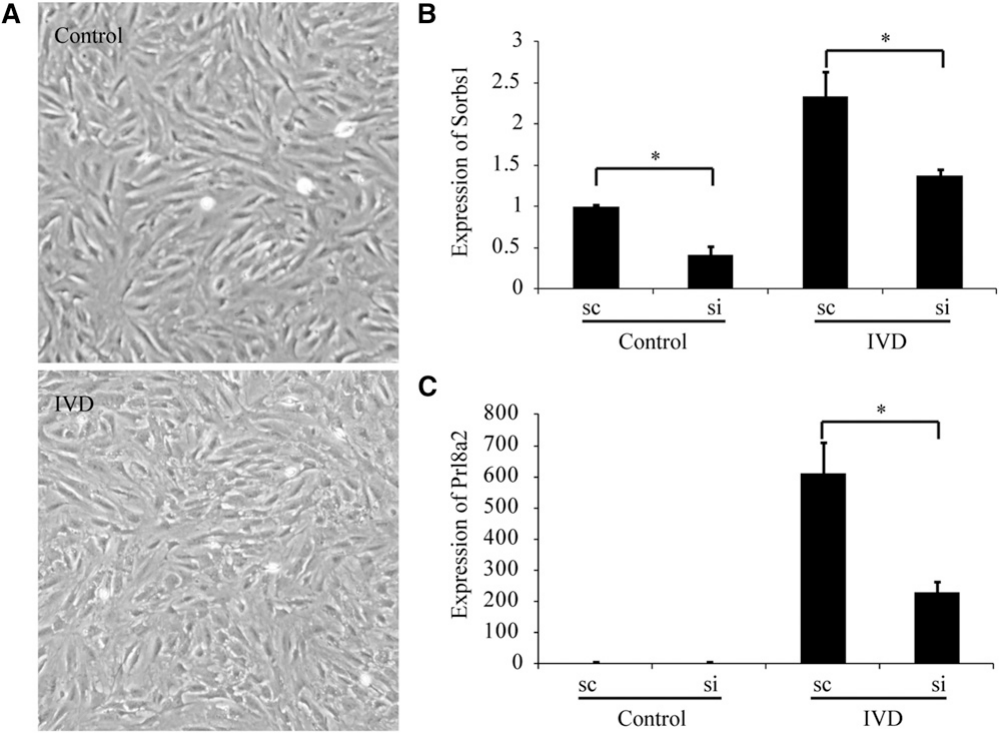
The involvement of *Sorbs1* in decidualization of mouse endometrial stromal cells. (A) The morphological changes in the cultured endometrial stromal cells upon *in vitro* decidualization (IVD). (B) Quantitative RT-PCR analysis of *Sorbs1* mRNA expression after endometrial stromal cells were transfected with *Sorbs1* targeting siRNA. (C) The influence of *Sorbs1* knockdown on the expression of decidualization marker gene *Prl8a2*. sc, scramble control; si, *Sorbs1* targeting siRNA. * *P* < 0.05.

On d 8 of pregnancy, stimulated by the invading embryo, the mouse uterus undergoes a rapid proliferation and differentiation process in the stromal compartment known as decidualization (Liu and Wang 2015). To investigate the role of *Sorbs1* during decidualization, isolated mouse endometrial stromal cells were induced for *in vitro* decidualization (Figure 5A). The expression of Sorbs1 was induced during *in vitro* decidualization (Figure 5B). We synthesized three siRNAs targeting *Sorbs1* and selected a most effective siRNA 5′-CCCUAUCACCUAUGUAGAUdTdT-3′, which was able to reduce *Sorbs1* expression by ∼ 60% (Figure 5B). Compared to scramble control, *Sorbs1*-targeting RNA significantly reduced the expression of decidualization marker gene *Prl8a2* on d 2 of *in vitro* decidualization (Figure 5C). These results demonstrated that *Sorbs1* contributes to decidualization of mouse endometrial stromal cells.

## Discussion

Despite the fact that genetic factors are believed to contribute largely to female infertility in humans, their role is only partially understood. Recently, the use of genome-wide association study has identified SNPs associated with lifetime number of pregnancies and number of children (Aschebrook-Kilfoy *et al.* 2015). However, all SNPs were nonsignificant after multiple test correction, indicating that female infertility is complex, and an individual SNP may exert only a modest effect. In the present study, we used inbred mouse strains to link female infertility to potential SNP sites. Our study may complement studies in humans.

Female infertility data, measured as the percent of matings that were nonproductive, were obtained from the Mouse Phenome Database at Jackson Laboratory. One clear limitation of this study is that the phenotyping was done in 1991, and only the average value for each mouse strain was given. Undoubtedly, the application of individual values will yield a more accurate result. Another limitation of this study is the complex composition of female infertility. The percent of matings that were nonproductive is a function of the number of corpora lutea, implantation rate, and middle pregnancy loss. Detailed phenotyping will add more information to the female infertility trait.

In the present study, we identified four significant SNPs after chromosome-wise multiple test correction. The first SNP rs29972765 is located on chr18:77022376 and its nearest gene is *Skor2* (SKI family transcriptional corepressor 2). In addition to *Skor2*, another five genes appeared to locate on the same LD block: *Ier3ip1* (immediate early response 3 interacting protein 1), *Hdhd2* (haloacid dehalogenase-like hydrolase domain containing 2), *Katnal2* (katanin p60 subunit A-like 2), *Pias2* (protein inhibitor of activated STAT 2), and *St8sia5* (ST8 alpha-N-acetyl-neuraminide alpha-2,8-sialyltransferase 5). To our knowledge, none of these genes are reported to play a role in female fertility. The second SNP rs30415957 resides on chr19:38799731 in the intron of *Plce1* (phospholipase C epsilon 1). Its LD block contained *Noc3l* (NOC3-like DNA replication regulator), *Tbc1d12* (TBC1 domain family member 12), and *Hells* (helicase lymphoid-specific). Of all genes in this LD block, *Hells* stands out because its downregulated expression in the endometrium is associated with repeated implantation failure (Koler *et al.* 2009) and recurrent pregnancy loss (Krieg *et al.* 2012; Ledee *et al.* 2011). However, the detailed role of *Hells* in female fertility has not been defined. The remaining two SNPs (rs30768258 and rs31216810) are close to each other in the same LD block on chromosome 19 in vicinity of *Sorbs1* (sorbin and SH3 domain containing 1).

Of all these genes, we focused on *Sorbs1*. A previous study has shown that *Sorbs1* is significantly upregulated at implantation sites compared to nonimplantation sites in mouse uterus on d 6.5 of pregnancy (Moreno-Moya *et al.* 2015). Using quantitative RT-PCR, we confirmed that the expression of *Sorbs1* is significantly upregulated at implantation sites compared to interimplantation sites on d 8 of pregnancy. The most remarkable event in the uterus on d 8 of pregnancy is decidualization, which is characterized as proliferation and subsequent differentiation of endometrial stromal cells into large epithelioid cells. To test whether *Sorbs1* plays a role in this process, we isolated mouse endometrial stromal cells and established a model of *in vitro* decidualization. We showed that expression of *Sorbs1* was induced during *in vitro* decidualization, mimicking the regulation in uterus on d 8 of pregnancy. Furthermore, we found that knockdown of *Sorbs1* by siRNA significantly reduced the expression of decidualization marker gene *Prl8a2* on day 2 of *in vitro* decidualization. Decidualization is required for uterine angiogenesis and hemostasis during trophoblast invasion and placenta formation (Blois *et al.* 2011), as well as the establishment of maternal immunological tolerance to embryonic antigens (Barrientos *et al.* 2014). Defects in decidualization during early pregnancy are associated with early pregnancy loss (Gellersen and Brosens 2014). Thus, *Sorbs1* may contribute to female fertility by exerting a role in decidualization. However, genes localized in the vicinity of the SNPs are not necessarily responsible for the trait (Smemo *et al.* 2014). Long-range interactions may exist. Therefore, further investigations, such as gene deletion and single-point genomic editing, may be needed to confirm the direct connection between *Sorbs1* and the female infertility trait.

In summary, we performed a genome-wide association study of female fertility in 25 inbred mouse strains by using publicly available SNP data. A total of four SNPs were identified, two of which are in vicinity of *Sorbs1*. Using an *in vitro* model of decidualization, we provided evidence that *Sorbs1* may be a potential candidate gene for female infertility in mice by modulating decidualization of endometrial stromal cells. Our results may represent an opportunity to further understand female infertility in humans.

## Supplementary Material

Supplemental Material
