# Supplementary material for: Genome-Wide Association Mapping for Female Infertility in Inbred Mice
Source: G3 (Bethesda). 2016 Jul 21;6(9):2929–35. doi: 10.1534/g3.116.031575 (PMC5015949; doi:10.1534/g3.116.031575)
Supplement: Supplemental Material [file supp_6_9_2929__index.html]

Genome-Wide Association Mapping for Female Infertility in Inbred Mice — Supplemental Material 

# Genome-Wide Association Mapping for Female Infertility in Inbred Mice

## Supplemental Material for Liu, *et al*, 2016

**Files in this Data Supplement:**

- Table S1 - A total of 158 SNPs significantly associated with female fertility in inbred mice (p < 0.001). (.xlsx, 24 KB)
